# Interaction of phosphorus and GA_3_ improved oilseed flax grain yield and phosphorus-utilization efficiency

**DOI:** 10.3389/fpls.2024.1435927

**Published:** 2024-08-01

**Authors:** Yingze Wang, Zhi Cao, Yuhong Gao, Bing Wu, Junyi Niu, Bin Yan, Yifan Wang, Zhengjun Cui, Ming Wen, Peng Xu, Haidi Wang, Xingkang Ma

**Affiliations:** ^1^ State Key Laboratory of Aridland Crop Science, Lanzhou, China; ^2^ College of Agronomy, Gansu Agricultural University, Lanzhou, China; ^3^ College of Life Science and Technology, Gansu Agricultural University, Lanzhou, Gansu, China; ^4^ College of Agronomy, Tarim University, Alaer, Xinjiang, China

**Keywords:** phosphorus, GA_3_, oilseed flax, phosphate fertilizer utilization, grain yield

## Abstract

**Introduction:**

Phosphorus nutrition and hormone concentration both affect crop yield formation. Ascertaining the interaction of phosphorus and GA_3_ has a synergistic effect on the grain yield and phosphorus utilization efficiency of oilseed flax in dryland. It is extremely important for improving grain yield and phosphorus utilization efficiency.

**Methods:**

A field experiment was conducted in 2019 and 2020 at the Dingxi Oil Crops Test Station to investigated the effects of phosphorus, gibberellin (GA_3_), and their interaction on the grain yield and phosphorus-utilization efficiency of oilseed flax plants. Phosphorus fertilizer was applied at three levels (0, 67.5, 135 kg P_2_O_5_·ha^-1^) and GA_3_ was also sprayed at three concentrations (0, 15, and 30 mg·L^-1^).

**Results:**

The results showed that application of 67.5 kg P_2_O_5_·ha^-1^ reduced leaves acid phosphatase (ACPase) activity, but increased phosphorus accumulation throughout the growth period, the 1000-kernel weight (TKW), and the number of grains per capsule. Spraying GA_3_ significantly increased the leaves ACPase activity, phosphorus accumulation after anthesis and its contribution to grain, phosphorus-utilization efficiency, the number of capsules per plant, and TKW. The phosphorus accumulation at the anthesis, kernel, and maturity stages under the treatment of fertilizing 67.5 kg P_2_O_5_·ha^-1^ and spraying 30 mg·L^-1^ GA_3_ were increased by 56.06%, 73.51%, and 62.17%, respectively, compared with the control (no phosphorus, no GA_3_). And the phosphorus accumulation after anthesis and its contribution to grain also increased. 67.5 kg P_2_O_5_·ha^-1^ combined with 30 mg·L^-1^ GA_3_ and 135 kg P_2_O_5_·ha^-1^ combined with 15 mg·L^-1^ GA_3_ both significantly increased grain yield of oilseed flax, reaching 1696 kg·ha^-1^ and 1716 kg·ha^-1^ across two years, respectively. And there was no significant difference between them. However, the former treatment significant increased the apparent utilization rate, agronomic utilization rate, and partial productivity of phosphorus. The interaction between phosphorus and GA_3_ was significant for grain yield.

**Conclusion:**

Therefore, the application of 67.5 kg P_2_O_5_·ha^-1^ in combination with 30 mg·L^-1^ GA_3_ is an effective fertilization approach for enhancing oilseed flax growth and grain yield in the experiment region and other similar areas.

## 1 Introduction

Phosphorus nutrition plays an important role in crop growth and development (Shahzado et al., 2016). The application of phosphorus fertilizer to farmland soil can not only promote plant growth, but also improve its absorption of mineral nutrients, which is conducive to yield formation (Shahzado et al., 2016). It is widely assumed that the phosphorus content of various vegetative organs increases as crops progress through the vegetative growth stage. And then gradually decreases during the reproductive growth stage, when most phosphorus is transferred to the grains, thus increasing crop yield (Fageria and Filho, 2007; Kakiuchi and Kamiji, 2015). However, crop yield and phosphorus absorption and accumulation differ among crop types and growth stages. According to previous studies, when phosphorus was applied within the range of 0–108 kg·ha^-1^, the yield of winter wheat (*Triticum aestivum* L.) increased with the increase of phosphorus application rate. But the yield and its component factors decreased when phosphorus was applied at rates higher than 108 kg·ha^-1^ (Zhu et al., 2012). Similar results have been obtained in studies on cotton (*Gossypium hirsutum* L.) (Wang et al., 2023), maize (*Zea mays* L.) (Fosu-Mensah and Mensah, 2016), and other crops. It has also been established that increasing the phosphorus application level reduces the phosphorus absorption efficiency and utilization rate, the phosphorus fertilizer recovery rate in the current season, and partial productivity (Ma et al., 2011; Zhu et al., 2012). Excessive phosphorus fertilizer input may disrupt the ecological balance among other nutrients instead of increasing crop yield. Therefore, for the long-term and sustainable development of agriculture, it is important to develop appropriate fertilization strategies to increase crop yield and phosphorus-utilization efficiency while reducing agricultural production costs. Many studies have demonstrated that exogenous hormones can increase the stress resistance and yield of crop plants (Falcioni et al., 2018). For example, gibberellic acid (GA) can enhance photosynthesis in leaves, and promote cell division, stem elongation, leaf expansion, and early flowering and fruit setting in crops. Consequently, it is widely used in agricultural production to improve crop yields and the quality of agricultural products (Falcioni et al., 2018). Previous researches have shown that spraying GA_3_ at the flowering stage can significantly increase TKW and yield of triticale (*Secale cereale* L.) (Yang et al., 2012). And it can also increase the number of effective panicles, grain plumpness, and carbohydrate transport and distribution from vegetative organs to grains in rice (*Oryza sativa* L.), thereby increasing the grain setting rate, TKW, and yield (Pan et al., 2013). Crop yield is heavily reliant on the absorption and utilization of nutrients and minerals from the soil. Various studies have demonstrated that spraying GA_3_ can increase the activity of enzymes involved in nitrogen metabolism, enhance light energy capture and CO_2_ assimilation, regulate carbon and nitrogen metabolism, promote nitrogen, phosphorus, and potassium accumulation (Bouton et al., 2002; Siddiqui et al., 2010). And it can also improve fertilizer utilization, and increase yield (Lin et al., 2016).

Oilseed flax (*Linum usitatissimum*. L) is one of the most important oil and cash crops in China, and it is also a phosphorus-sensitive crop (Xie et al., 2014b). A suitable phosphorus application can significantly improve the phosphorus transport capacity, transport rate, and the contribution rate to oilseed flax grain, and significantly increase the effective number of capsules per plant, grain number per capsule, and TKW, thereby increasing yield (Xie et al., 2014b). However, as crops’ phosphorus consumption increases, phosphorus absorption efficiency, phosphorus-utilization efficiency, seasonal recovery rate of phosphorus, and the partial productivity of phosphorus decreased accordingly. This not only raises agricultural production costs, but also depletes limited phosphate rock resources and causes environmental pollution (Grant et al., 2009; Khan et al., 2018). It has been shown that GA_3_ can boost the activity of nitrogen metabolism enzymes and increase the nitrogen-utilization efficiency of oilseed flax plants (Khan and Mohammad, 2013). Under low-phosphorus conditions, exogenous GA_3_ can increase the surface area for nutrient absorption by increasing the number of cells in the root elongation zone, thereby improving phosphorus absorption (Zhang et al., 2019). In plants with a sufficient nitrogen supply, spraying GA_3_ can significantly improve their nitrogen-use efficiency (Khan et al., 2002). Thus, different nutrients and exogenous GA_3_ have synergistic effects on crop yield formation and fertilizer utilization. However, the effects of phosphorus fertilizer and GA_3_ and their interactions on phosphorus utilization and yield formation on oilseed flax have not been reported yet. Therefore, the aim of this study was to investigate the effects of different concentrations of phosphorus fertilizer and exogenous GA_3_ on leaf acid phosphatase (ACPase) activity, phosphorus accumulation and transport, grain yield, and the phosphorus-utilization efficiency of oilseed flax. We hypothesized that the interaction between phosphorus and GA_3_: 1. would enhance ACPase activity in dryland oilseed flax leaves, 2. could increase phosphorus accumulation, promote phosphorus transport to grain, and thus improve the phosphorus fertilizer-utilization efficiency of oilseed flax, 3. might increase TKW and the number of grains per capsule, thereby promoting grain yield of oilseed flax. The specific objectives of this study were as follows: (1) To investigate the effects of the phosphorus × GA_3_ interaction on phosphorus accumulation and transport in oilseed flax plants; (2) to devise an efficient fertilization strategy to improve the phosphorus-utilization efficiency and grain yield of oilseed flax.

## 2 Materials and methods

### 2.1 Experimental site

**Figure 1:**
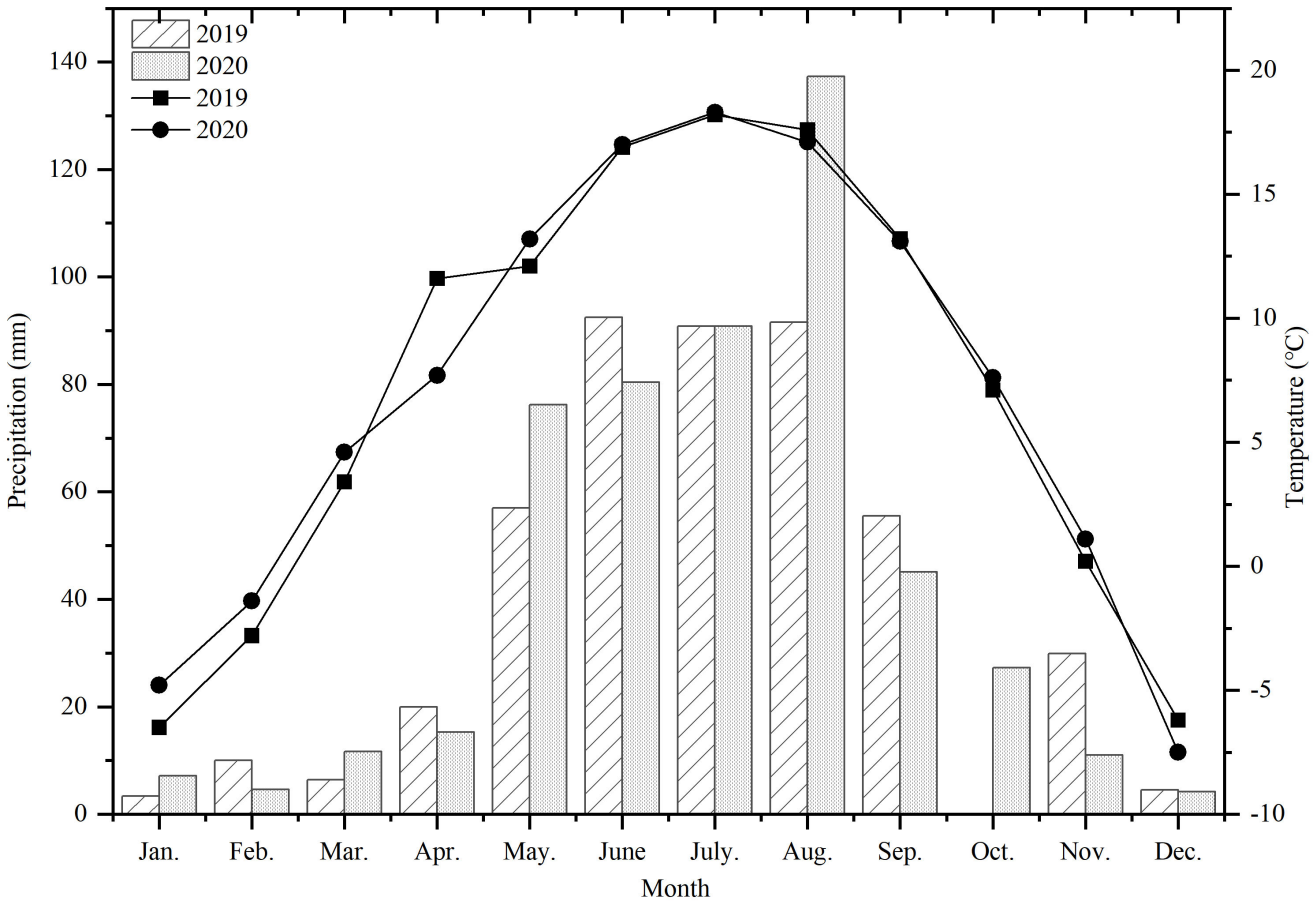
Average monthly precipitation and temperature in 2019 and 2020.

From March 2019 to August 2020, a field experiment was carried out at the Institute of Oil Crops, Dingxi Academy of Agricultural Sciences (34°26′N, 103°52′E), which is located in the semi-arid hilly and gully area of the Loess Plateau in the middle of Gansu Province, China. The average elevation of the site is 2,050 m above sea level, the average annual temperature is 6.43 °C, the average annual sunshine hours is 2453, the frost-free period is 140 days, and the average annual rainfall is approximately 486.3 mm. The climate details for this experiment are shown in **Figure 1**, in which it can be seen that 2019 is a normal flow year, and 2020 is a high flow year, with a rainfall of 511.1 mm. The test area was a terraced field with loess soil of equal fertility, and the soil bulk density is 1.35 g·cm^-3^. The average values of the major nutrients in the 0–30 cm soil layer in 2019 and 2020 were as follows: organic matter, 17.51 g·kg^-1^; total nitrogen, 0.81 g·kg^-1^; total phosphorus, 0.69 g·kg^-1^; available phosphorus, 27.43 mg·kg^-1^; available potassium, 108.30 mg·kg^-1^; pH, 8.14.

### 2.2 Experimental design and plot management

The field experiment had a two-factor split plot design, with the main factor being the amount of phosphorus fertilizer applied (as pure P_2_O_5_) and the secondary factor being the foliar spreading concentration of GA_3_. The phosphorus fertilizer was applied at three levels: 0 kg·ha^-1^ (P_0_), 67.5 kg·ha^-1^ (P_1_), and 135 kg·ha^-1^ (P_2_). And GA_3_ was also sprayed at three concentrations: 0 mg·L^-1^ (G_0_), 15 mg·L^-1^ (G_1_), and 30 mg·L^-1^ (G_2_). There were nine treatments in total, with P_0_G_0_ as the control. Each treatment had three replicates. The plots were 6 m long and 3 m wide. The subplots were 3 m long and 2 m wide, the distance between replicates was 80 cm, and a 1-m protection row was installed around the test site. The test site was 28 m long and 13 m wide (364 m^2^ in total).

The oilseed flax variety used in this study was ‘Lunxuan No. 2’, which was provided by the Inner Mongolia Academy of Agricultural and Animal Husbandry Science. The sowing rate was 7.5 million seeds·ha^-1^, the sowing depth was 3 cm, and the row spacing was 20 cm. The final seedling density was 3.75 million plants·ha^-1^. In each treatment, in addition to applying phosphorus as described above as superphosphate (P_2_O_5_, 16%), nitrogen fertilizer was applied at 150 kg N·ha^-1^ (urea, N 46%) and potassium sulfate was applied at 52.5 kg·ha^-1^ (K_2_O, 52%). Both were applied as base fertilizer. For the GA_3_ spraying treatment, the oilseed flax plants were sprayed at the budding stage and anthesis stage with 1 L GA_3_ solution (G_1_ and G_2_) or clean water (G_0_) per treatment. The oilseed flax seeds were sown on April 10th (2019) and April 9th (2020) and the mature plants were harvested on August 31st (2019) and August 19th (2020). Other management methods were as per general field.

### 2.3 Measurements and calculations

#### 2.3.1 Determination of ACPase activity

In 2019 and 2020, 30 representative plant samples with consistent growth were collected from each plot before spraying GA_3_ and at 1, 3, 5 and 10 days after spraying GA_3_ at the budding stage and the anthesis stage. The middle and upper leaves were rapidly frozen in liquid nitrogen and then stored at −80 °C until analysis. After collecting all the samples, an enzyme extract was prepared from the oilseed flax leaves and ACPase activity was determined using a kit from the Suzhou Cemin Biotechnology Co., Ltd., Suzhou, China. The absorbance of the reaction solution was measured with a microplate reader.

#### 2.3.2 Determination of phosphorus content in plant tissues

At each growth stage of oilseed flax, the plants were divided into stems, leaves, flowers, buds, capsules, and other organs, which are killed in a 105 °C incubator for 30 minutes. Then, the temperature was dropped to 80 °C and dried for 6-8 hours until constant weight. The dry weight of each organ was measured and the accumulation of dry matter was calculated.

The dried samples were crushed in a stainless-steel cyclone mill (Dewei, 100T, Guangzhou, China), digested in concentrated H_2_SO_4_-H_2_O_2_, and the phosphorus content was measured by vanadium molybdenum yellow colorimetry. The formulae used to calculate phosphorus accumulation, transportation, and utilization in oilseed flax plants were as follows (Wu et al., 2016):

Phosphorus accumulation (kg·ha^-1^) = dry matter weight × phosphorus content in plant tissue

Phosphorus transport capacity of vegetative organs (kg·ha^-1^) = phosphorus accumulation in stems and leaves at the anthesis stage − phosphorus accumulation in stems and leaves at the maturity stage

Phosphorus input into grains after anthesis (kg·ha^-1^) = phosphorus accumulation in grains at maturity – amount of phosphorus transported from vegetative organs

Phosphorus contribution rate (%) = amount of phosphorus transported/amount of phosphorus accumulated in grain at maturity × 100%

Agricultural phosphorus fertilizer utilization rate (kg·kg^-1^) = (yield from area where phosphorus fertilizer was applied − yield from area where no phosphorus fertilizer was applied)/amount of phosphorus fertilizer applied

Phosphorus fertilizer apparent utilization rate (%) = (phosphorus taken up by aboveground plant parts in area where phosphorus fertilizer was applied − phosphorus taken up by aboveground plant parts in area where no phosphorus fertilizer was applied)/amount of phosphorus fertilizer applied × 100%

Phosphorus fertilizer partial productivity (kg·kg^-1^) = grain yield/amount of phosphorus fertilizer applied

#### 2.3.3 Determination of major nutrients in the 0–30 cm soil layer

Organic matter was determined by potassium dichromate method, total nitrogen was determined by semi micro Kjeldahl method, total phosphorus and available phosphorus were determined by molybdenum antimony resistance colorimetry, total potassium and available potassium were determined by FP6410 flame photometer, and pH was determined by pH meter (Bao, 2000).

#### 2.3.4 Determination of yield and yield components

At the maturity stage, 30 plants with consistent levels of maturity were randomly selected from each plot for seed testing. The number of capsules per plant, the number of grains per capsule, and TKW were recorded. At harvest, the oilseed flax grain weight was measured after drying at 25°C for 30 h, and the actual grain yield per plot was calculated.

### 2.4 Statistical analysis

Each measurement was conducted with three replicates. The effects of different treatments on the measured variables were determined by ANOVA. The data were analyzed using F-test, and multiple comparisons were performed using the least significant difference test (LSD) (*P* ≤ 0.05). The experimental data were analyzed with the SPSS statistical package v.24.0 (SPSS Inst., Cary, NC, USA) and the figures were generated using Origin 2021 (Systat Software Inc.).

## 3 Results

### 3.1 Effects of phosphorus and exogenous GA_3_ on acid phosphatase activity in oilseed flax leaves

#### 3.1.1 ACPase activity at the budding stage

**Figure 2:**
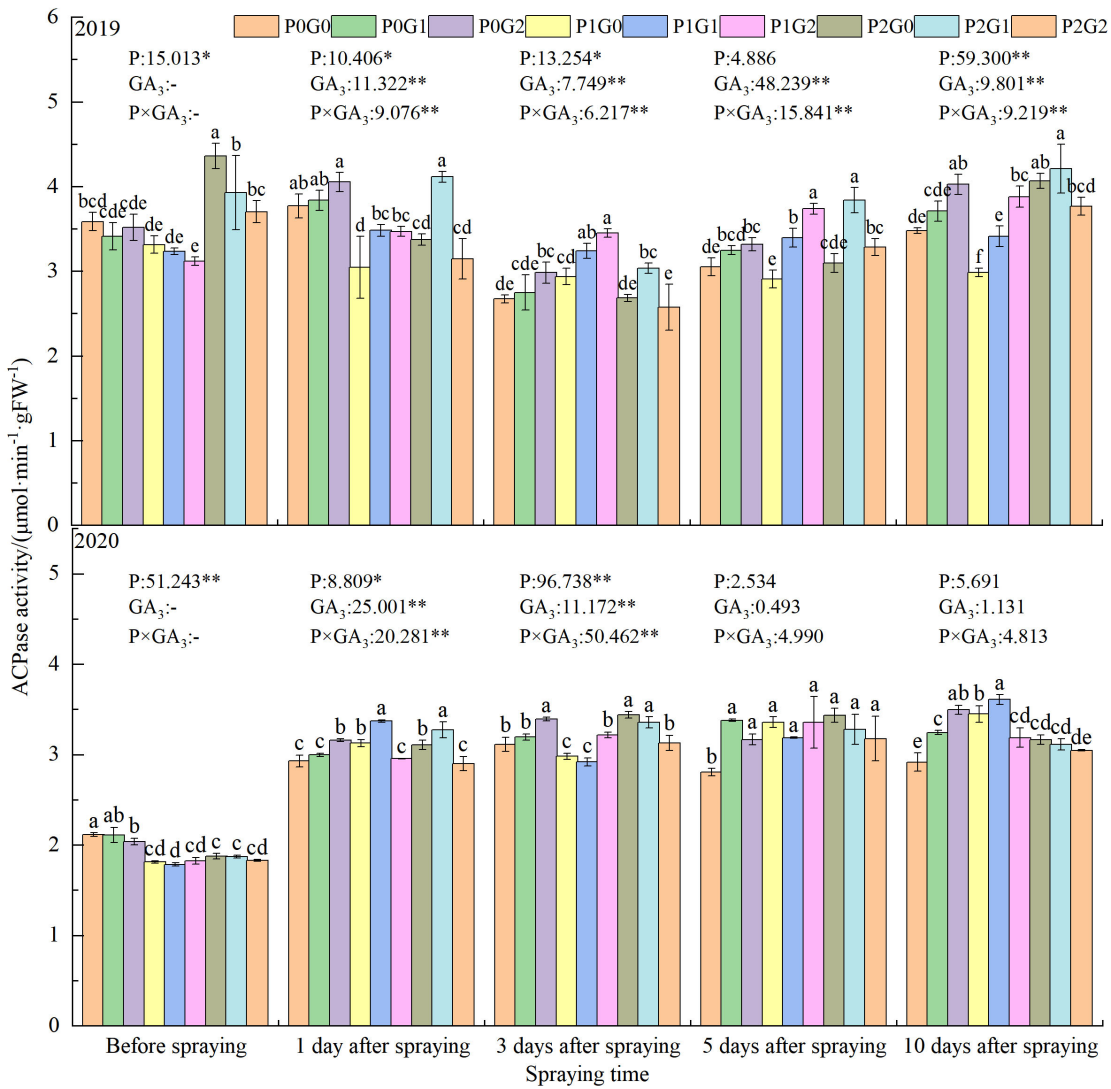
Effects of phosphorus application levels and GA_3_ concentrations on leaves ACPase activity in oilseed flax plants at the budding stage. Different small letters indicate significant difference among treatments (*P<*0.05). * indicate significant difference at 0.05 level, ** indicate highly significant difference at 0.01 level; the number before * represents the coefficient of variation; “-” indicate no GA_3_ spray.

The leaves ACPase activity was significantly affected by the amount of phosphorus application, the concentration of GA_3_, and the interaction between these two factors (P×GA_3_) (**Figure 2**). In both growth seasons, before spraying GA_3_, the ACPase activity of oilseed flax leaves was the lowest in the P_1_ treatments. The leaves ACPase activity in the P_1_ treatments was significantly lower than that in the P_0_ treatments (by 8.09% in 2019 and by 13.41% in 2020) and the P_2_ treatments (by 24.08% in 2019 and by 2.77% in 2020).

At the first day after spraying GA_3_, the leaves ACPase activity was 8.63% higher in the G_1_ treatments than G_0_ treatments. The interaction between phosphorus and GA_3_ also significantly affected leaves ACPase activity. Compared with the leaves ACPase activity in the G_0_ treatments (no GA_3_), that in P_1_G_1_ with and P_2_G_1_ was increased by 7.78%–14.27% and 5.38%–21.91%, respectively. In the P_1_ treatments, P_1_G_2_ had the highest ACPase activity at 3 to 5 days after spraying GA_3_, which was 9.72%–12.03% (3 days) and 2.66%–19.35% (5 days) higher than that in P_1_G_1_ and P_1_G_0_, respectively. Within each GA_3_ concentration, the ACPase activity in oilseed flax leaves varied with the amount of phosphorus fertilizer application. At 1–5 days after spraying GA_3_, the leaves ACPase activity was 3.04%–12.77% higher (P_2_G_0_) and 3.51%–18.56% higher (P_2_G_1_) than that in the P_0_ treatments, but it increased in P_0_G_2_ by 10 days after spraying GA_3_. These results showed that a low phosphorus fertilizer level combined with a high concentration of GA_3_ as a foliar spray (P_1_G_2_) could synergistically improve leaves ACPase activity at the budding stage of oilseed flax.

#### 3.1.2 ACPase activity at anthesis stage

**Figure 3:**
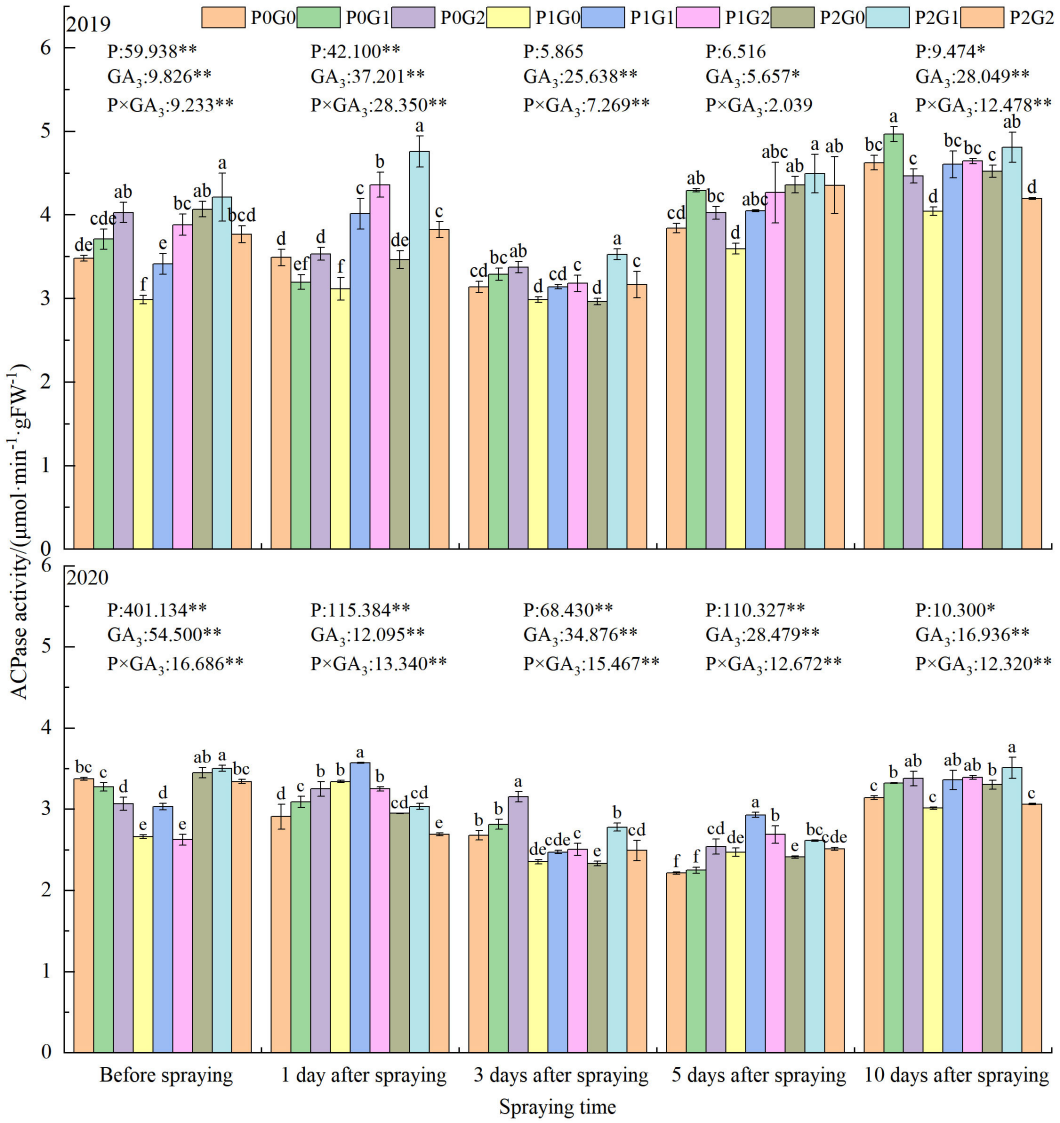
Effects of phosphorus application levels and GA_3_ concentrations on leaves ACPase activity at anthesis stage. Different small letters indicate significant difference among treatments (*P<*0.05). * indicate significant difference at 0.05 level, ** indicate highly significant difference at 0.01 level; the number before * represents the coefficient of variation; “-” indicate no GA_3_ spray.

The phosphorus level, GA_3_ concentration, and P×GA_3_ had significant effects on leaves ACPase activity at the anthesis stage (**Figure 3**). Before spraying GA_3_, the ACPase activity in leaves was the highest in the P_2_ treatments, where it was 5.94%–7.42% and 17.19%–23.78% higher than that in the P_0_ and P_1_ treatments, respectively. At 1 day after spraying GA_3_, the ACPase activity was significantly higher in the P_1_ treatments (by 9.88%–12.47%) than that in the P_0_ treatments. At 3 d and 10 d after spraying GA_3_, it was higher in the P_0_ treatments than that in the P_1_ treatments. In the P_0_ and P_1_ treatments, the higher concentration of GA_3_ (G_2_) increased the leaves ACPase activity at 1–3 days and 10 days after spraying GA_3_, to 1.21%–17.69% and 12.51%–14.80% higher, respectively, than that in the G_0_ treatments. In the P_2_ treatments, the lower concentration of GA_3_ (G_1_) significantly increased the leaves ACPase activity at 1–10 days after spraying in the two growth seasons. Spraying GA_3_ significantly increased the leaves ACPase activity by 3.06%–37.41% and 2.82%–35.94% in 2019 and 2020, respectively, compared with that in the G_0_ treatments.

In terms of the GA_3_ concentration, the leaves ACPase activity in the G_1_ treatments was the highest at 1–10 days after spraying GA_3_, and was 7.01%–18.85% (*P*<0.05) and 5.40%–9.78% (*P*<0.05) higher than that in the G_0_ treatments in 2019 and 2020, respectively. At 1, 5, and 10 days after spraying GA_3_, the leaves ACPase activity in the P_1_ treatments was higher than that in the G_2_ treatments, and was 5.96%–23.42% higher than that in the G_0_ treatments. At 3 and 10 days after spraying GA_3_, the leaves ACPase activity in P_2_G_1_ was 5.61%–12.76% higher than that in the P_0_ treatments. Across the two growing seasons, the ACPase activity of oilseed flax leaves in P_2_G_1_ was 12.42%, 15.47%, and 7.80% higher than that in P_0_G_0_ before spraying GA_3_, and at 3 and 10 days after spraying GA_3_, respectively. Thus, the combination of 135 kg P_2_O_5_·ha^-1^ as fertilizer and 15 mg·L^-1^ GA_3_ as a foliar spray significantly increased the ACPase activity of oilseed flax leaves at the anthesis stage, thereby increasing phosphorus accumulation in the plants.

### 3.2 Effects of phosphorus and exogenous GA_3_ on phosphorus accumulation in oilseed flax plants

**Figure 4:**
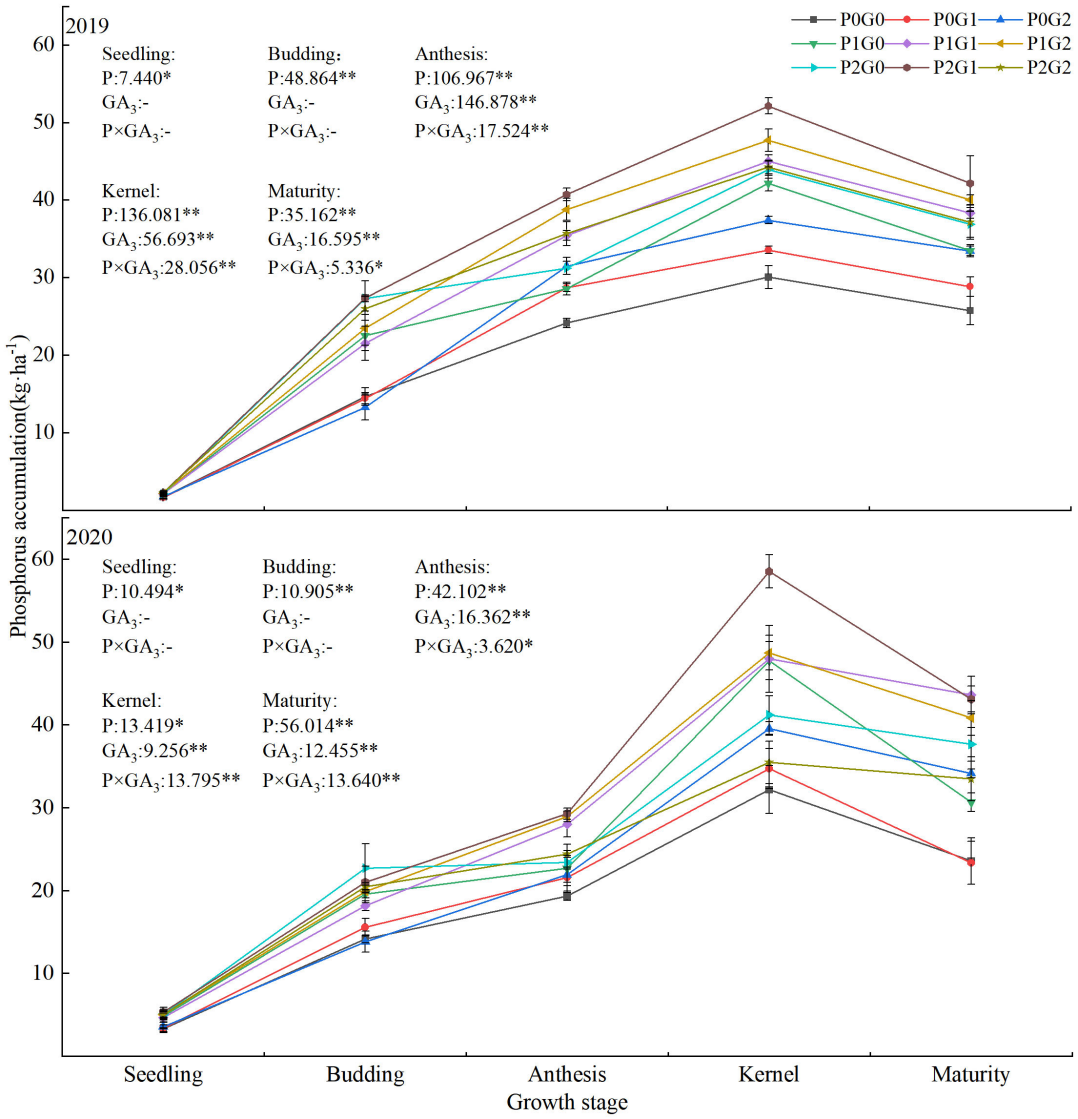
Phosphorus accumulation in oilseed flax plants under different phosphorus fertilizer levels and GA_3_ concentrations. * indicate significant difference at 0.05 level, ** indicate highly significant difference at 0.01 level; the number before * represents the coefficient of variation; “-” indicate no GA_3_ spray.

During the growth of oilseed flax plants, phosphorus accumulation increased first and then decreased, reaching the maximum value at the kernel stage (**Figure 4**). The amount of phosphorus application significantly affected phosphorus accumulation during the whole oilseed flax growth period. The application of GA_3_ had a highly significant effect on phosphorus accumulation from the anthesis stage to the maturity stage. The effect of the interaction between phosphorus and GA_3_ on the phosphorus accumulation from anthesis to maturity stage also reached a significant level. At the seedling and budding stages, phosphorus accumulation in the P_1_ and P_2_ treatments was 26.35%–41.78% and 30.35%–50.39% (seedling stage) and 32.40%–59.44% and 47.55%–90.72% (budding stage) higher than that in the P_0_ treatments, respectively. From anthesis to the maturity stage, phosphorus accumulation in the P_1_ and P_2_ treatments was 4.70%–42.01% higher than that in the P_0_ treatments, and 15.06%–26.19% higher in the G_1_ and G_2_ treatments than that in the G_0_ treatments. In the P_0_ and P_1_ treatments, phosphorus accumulated to the highest levels in P_0_G_2_ and P_1_G_2_. Within each GA_3_ treatment, phosphorus accumulated to higher levels in the P_1_ and P_2_ treatments than that in the P_0_ treatments. The treatment with the highest phosphorus accumulation from the seedling to maturity stage was P_2_G_1_ (2.25–58.53 kg·ha^-1^) followed by P_1_G_2_ (2.16–48.70 kg·ha^-1^). From anthesis to the maturity stage, across the two growing seasons, phosphorus accumulation in P_2_G_1_ and P_1_G_2_ was 51.27%–83.11% and 49.61%–73.38% higher, respectively, than that in P_0_G_0_. These results showed that a low level of phosphorus fertilizer combined with a high concentration of GA_3_ as a foliar spray, or a high level of phosphorus fertilizer combined with a low concentration of GA_3_ as a foliar spray, synergistically increased phosphorus accumulation of oilseed flax.

### 3.3 Effects of phosphorus and exogenous GA_3_ on phosphorus transport in oilseed flax plants

The phosphorus level significantly affected phosphorus transfer before anthesis, phosphorus accumulation after anthesis, and the contribution rate to grains in both two growing seasons. Spraying GA_3_ and P×GA_3_ also had significant effects on phosphorus accumulation after anthesis and its contribution to grain in both growing seasons (**Table 1**). Across the two growing seasons, the amount of phosphorus transferred before anthesis was significantly higher in the P_0_ treatments than that in the P_1_ and P_2_ treatments (by 17.35%-85.26% and 14.85%-106.57%, respectively). And its contribution rate to grains was also significantly higher in the P_0_ treatments than that in the P_1_ and P_2_ treatments (by 30.80%–111.83% and 30.80%-139.82%, respectively). Spraying GA_3_ significantly reduced the contribution rate of pre-anthesis phosphorus transfer to grain in both growing seasons.

**Table 1:** Phosphorus transfer before anthesis, phosphorus accumulation after anthesis, and their contribution to grains under different treatments.

Treatment	Phosphorus transfer before anthesis (kg·ha^-1^)		Phosphorus accumulation after anthesis (kg·ha^-1^)		Contribution before anthesis to grains (%)		Contribution after anthesis to grains (%)	
	2019	2020	2019	2020	2019	2020	2019	2020
P_0_G_0_	10.70 ± 0.43a	8.15 ± 0.11a	6.45 ± 0.58g	14.24 ± 0.49e	62.28a	36.44a	37.72f	63.56d
P_0_G_1_	10.65 ± 0.32a	8.20 ± 0.18a	7.52 ± 0.53f	16.19 ± 0.85d	58.86b	33.80b	41.14e	66.20c
P_0_G_2_	10.48 ± 0.65a	8.40 ± 0.16a	9.09 ± 0.71e	17.42 ± 0.27d	53.77c	32.59b	46.23d	67.41c
P_1_G_0_	6.19 ± 0.32b	8.11 ± 0.20a	12.29 ± 0.42d	16.20 ± 0.18d	33.57d	33.42b	66.43c	66.58c
P_1_G_1_	5.54 ± 0.41c	6.44 ± 0.09c	16.03 ± 0.72b	21.39 ± 0.50b	25.78e	23.20d	74.22b	76.80a
P_1_G_2_	5.45 ± 0.34c	6.54 ± 0.03c	17.53 ± 0.89a	23.16 ± 0.56a	23.78ef	22.08d	76.22ab	77.92a
P_2_G_0_	5.15 ± 0.46c	7.64 ± 0.11b	15.60 ± 0.66b	19.23 ± 0.33c	24.85ef	28.48c	75.15ab	71.52b
P_2_G_1_	5.05 ± 0.40c	6.44 ± 0.27c	17.65 ± 1.37a	22.61 ± 1.65ab	22.34f	22.23d	77.66a	77.77a
P_2_G_2_	5.20 ± 0.16c	7.48 ± 0.07b	14.86 ± 0.64c	19.03 ± 0.82c	25.96e	28.26c	74.04b	71.74b
Significance (*P* value)								
P	53.682**	17.282**	70.775**	43.075**	49.915**	19.635**	114.870**	107.029**
GA_3_	3.330	44.109**	39.125**	84.012**	3.205	65.531**	19.226**	212.086**
P×GA_3_	2.919	16.234**	12.947**	20.916**	1.946	18.095**	3.560*	39.404**

Different small letters indicate significant difference among treatments (P<0.05). * indicate significant difference at 0.05 level, ** indicate highly significant difference at 0.01 level, the number before * represents the coefficient of variation.

Phosphorus accumulation after anthesis increased with increasing GA_3_ concentration in the P_0_ and P_1_ treatments. In the P_0_ and P_1_ treatments, phosphorus accumulation after anthesis was 22.33%-40.93% higher and 42.64%-42.96% higher, respectively, in the G_2_ treatments than that in the G_0_ treatments. Phosphorus accumulation after anthesis was 13.14%-17.58% higher in P_2_G_1_ than that in P_2_G_0_, and 32.95%–92.85% higher in P_2_G_1_ than that in P_0_G_0_ and P_0_G_1_. In the P_0_ and P_1_ treatments, the GA_3_ treatments were ranked, from largest to smallest contribution rate of phosphorus accumulated post-anthesis to grain phosphorus, as follows: G_2_>G_1_>G_0_. In the G_0_ and G_1_ treatments, the rank order was P_2_>P_1_>P_0_. The rate in P_2_ was significantly higher than that in P_0_ by 12.52%-99.23% (in G_0_) and 17.48%-88.77% (in G_1_).

The results showed that phosphorus fertilizer application combined with spraying GA_3_ promoted phosphorus accumulation after anthesis. In particular, the combinations of a low level of phosphorus with a high GA_3_ concentration (P_1_G_2_) and a high phosphorus fertilizer level with a low GA_3_ concentration (P_2_G_1_) significantly promoted phosphorus accumulation after anthesis and its contribution to grain. In the P_1_G_2_ and P_2_G_1_ treatments, phosphorus accumulation after anthesis was 62.64%-171.78% and 58.77%-173.64% higher, respectively, than that in P_0_G_0_. And the contribution rate of phosphorus accumulation after anthesis to grain was 22.59%-102.67% and 22.36%-105.89% higher, respectively, than that in P_0_G_0_, with no significant difference between them. Thus, application of 67.5 kg P_2_O_5_·ha^-1^ as fertilizer combined with 30 mg·L^-1^ GA_3_ and 135 kg P_2_O_5_·ha^-1^ combined with 15 mg·L^-1^ GA_3_ were beneficial in terms of phosphorus accumulation and its contribution to grain after anthesis, thereby increasing grain yield.

### 3.4 Effects of phosphorus and exogenous GA_3_ on oilseed flax yield and its components

The phosphorus level, GA_3_ concentration, and P×GA_3_ had significant effects on TKW and grain yield of oilseed flax (**Table 2**). Compared with the P_0_ treatments, the P_1_ and P_2_ treatments significantly increased the grain yield by 9.20%-10.13% and 10.33%-11.27%, respectively, and TKW by 1.00%-3.96% and 3.68%-5.48%, respectively. Under different phosphorus fertilization levels, grain yield and TKW responded differently to GA_3_ concentrations. In the P_0_ and P_1_ treatments, the grain yield increased as the GA_3_ concentration increased, and TKW showed the same trend in 2020. In the P_2_ treatments, the grain yield and TKW were higher in the G_1_ treatment than that in the other GA_3_ treatments by 2.91%-8.66% and 2.91%-11.69%, respectively (*P*<0.05). The amount of phosphorus fertilizer had a significant impact on the grain number per capsule, and was significantly higher in the P_2_ treatments (by 5.53%-13.19%) than that in the P_0_ treatments. The effective number of capsules per plant was 9.28%-11.07% higher in the G_1_ treatments and 5.45%-7.98% higher in the G_2_ treatments than that in the G_0_ treatments across the two growing seasons. The phosphorus fertilizer treatments were ranked, from the highest to the lowest grain yield, TKW, and grain number per capsule, as follows: P_2_>P_1_>P_0_ (in the G_0_ and G_1_ treatments) and P_1_>P_2_>P_0_ (in the G_2_ treatment). In conclusion, both P_1_G_2_ and P_2_G_1_ treatments increased the capsule number per plant, grain number per capsule, and TKW, thereby increasing the grain yield of oilseed flax. Thus, these strategies are suitable for optimizing the grain yield of oilseed flax in this area.

**Table 2:** Grain yield and yield components of oilseed flax under different treatments.

Year	Treatment	Grain yield (kg·ha^-1^)	Capsule number per plant	Grain number per capsule	1000-kernel weight (g)
2019	P_0_G_0_	1 446 ± 15.41e	17.30 ± 0.67d	6.23 ± 0.21d	6.54 ± 0.08c
	P_0_G_1_	1 491 ± 11.09d	20.73 ± 0.82c	7.41 ± 0.29abc	6.75 ± 0.04bc
	P_0_G_2_	1 507 ± 6.34d	21.53 ± 0.93bc	7.08 ± 0.23c	6.72 ± 0.14c
	P_1_G_0_	1 548 ± 19.94c	21.98 ± 1.41bc	7.11 ± 0.26bc	6.70 ± 0.04c
	P_1_G_1_	1 621 ± 13.72b	22.40 ± 0.43bc	7.75 ± 0.54abc	7.05 ± 0.11b
	P_1_G_2_	1 688 ± 9.29a	21.97 ± 0.45bc	7.70 ± 0.22abc	7.05 ± 0.13b
	P_2_G_0_	1 566 ± 9.98c	22.88 ± 0.27b	7.85 ± 0.49ab	6.76 ± 0.07bc
	P_2_G_1_	1 701 ± 10.66a	24.80 ± 0.16a	8.11 ± 0.23a	7.55 ± 0.12a
	P_2_G_2_	1 640 ± 39.97b	22.05 ± 1.47bc	7.50 ± 0.08abc	6.80 ± 0.29bc
2020	P_0_G_0_	1 481 ± 15.12f	18.92 ± 0.62cd	7.43 ± 0.35b	6.45 ± 0.02f
	P_0_G_1_	1 531 ± 11.34e	19.67 ± 0.85bcd	7.45 ± 0.04b	6.74 ± 0.01e
	P_0_G_2_	1 546 ± 7.56e	20.94 ± 0.17bcd	7.73 ± 0.01b	6.85 ± 0.03cd
	P_1_G_0_	1 583 ± 7.56d	17.93 ± 0.23d	7.56 ± 0.36b	6.49 ± 0.02f
	P_1_G_1_	1 654 ± 10.59c	21.23 ± 0.71ab	7.80 ± 0.08ab	6.75 ± 0.01e
	P_1_G_2_	1 704 ± 15.12ab	20.76 ± 0.17abc	7.81 ± 0.34ab	7.00 ± 0.01b
	P_2_G_0_	1 593 ± 15.12d	19.67 ± 1.52bcd	7.71 ± 0.03b	6.88 ± 0.01c
	P_2_G_1_	1 731 ± 7.56a	21.88 ± 0.09a	8.25 ± 0.12a	7.08 ± 0.04a
	P_2_G_2_	1 682 ± 12.85b	19.33 ± 1.44bcd	7.90 ± 0.16ab	6.81 ± 0.04e
Significance (*P* value)					
2019	P	155.958**	21.273**	66.097**	27.831**
	GA_3_	46.846**	7.314**	6.865*	14.488**
	P×GA_3_	7.649**	5.627**	2.161	4.703*
2020	P	173.686**	2.113	36.469**	264.736**
	GA_3_	125.251**	7.222**	2.255	182.010**
	P×GA_3_	14.360**	2.766	0.963	72.438**

Different small letters indicate significant difference among treatments (P<0.05). * indicate significant difference at 0.05 level, ** indicate highly significant difference at 0.01 level, the number before * represents the coefficient of variation.

### 3.5 Correlation coefficients and general coefficients of oilseed flax grain yield and yield components

There were significant positive correlations between grain yield and grain number per capsule, as well as grain yield and TKW (**Table 3**). In 2019, the yield components were ranked, from the largest contribution to the smallest to grain yield, as follows: TKW > grain number per capsule > capsule number per plant. In 2020, the rank order was grain number per capsule > TKW > capsule number per plant. These results showed that optimal phosphorus fertilizer levels and foliar GA_3_ treatment positively affected the two key yield components, grain number per capsule and TKW. The indirect path analysis revealed that in 2019, capsule number per plant and grain number per capsule had a greater impact on grain yield *via* their effects on TKW, while TKW primarily affected the grain yield *via* grain number per capsule. Capsule number per plant had a significant impact on grain yield in 2020. Grain number per capsule mainly affected the grain yield via TKW. And TKW affected the grain yield *via* grain number per capsule. These results reveal that the effects of yield components on grain yield were mainly achieved through the indirect effects of capsule number per plant and grain number per capsule, as well as TKW.

**Table 3:** Correlation and passage coefficients of oilseed flax grain yield and yield components.

Year	Yield components	Correlation coefficient with yield	Direct path coefficient	Indirect path coefficient		
				Capsule number per plant	Grain number per capsule	1000-kernel weight
2019	Capsule number per plant	0.781**	0.047	―	0.347	0.386
	Grain number per capsule	0.809**	0.378	0.043	―	0.387
	1000-kernel weight	0.831**	0.505	0.036	0.289	―
2020	Capsule number per plant	0.548	-0.274	―	0.597	0.225
	Grain number per capsule	0.878**	0.848	-0.193	―	0.223
	1000-kernel weight	0.735*	0.282	-0.219	0.672	―

* indicate significance at 0.05 level, ** indicate highly significance at 0.01 level, the number before * represents the coefficient of variation.

### 3.6 Effects of phosphorus and exogenous GA_3_ on phosphorus-utilization efficiency of oilseed flax

The phosphorus level, GA_3_ concentration, and P×GA_3_ significantly affected the apparent utilization rate, agronomic utilization rate, and partial productivity of oilseed flax in the two growing seasons (**Table 4**). The apparent utilization rate, agronomic utilization rate, and partial productivity of oilseed flax were 44.33%, 49.41%, and 47.80% higher, respectively, in the P_1_ treatments than that in the P_2_ treatments. In the P_1_ treatments, the apparent utilization rate, agronomic utilization rate, and partial productivity of phosphate fertilizer all increased with increasing GA_3_ concentration, and they were 85.36%-143.25%, 118.54%-137.75%, and 7.59%-9.03% higher, respectively, in the G_2_ treatments than that in the G_0_ treatments. In the P_2_ treatments, the apparent utilization rate, agronomic utilization rate, and partial productivity of phosphate fertilizer were 38.66%-47.69%, 112.35%-123.00%, and 8.62%-8.64% higher, respectively, in the treatments G_1_ than that in the G_0_ treatments. Within each GA_3_ level, the apparent utilization efficiency, agronomic utilization efficiency, and partial productivity of phosphorus fertilizer decreased as the amount of phosphorus fertilizer application increased. Overall, the treatment combining 67.5 kg P_2_O_5_·ha^-1^ as fertilizer and 30 mg·L^-1^ GA_3_ as a foliar spray significantly improved the phosphorus fertilizer-utilization efficiency of oilseed flax and reduced phosphorus fertilizer losses.

**Table 4:** Changes of phosphorus-utilization efficiency of oilseed flax under different treatments.

Treatment	Apparent utilization rate (%)		Agronomic utilization rate (kg·kg^-1^)		Partial productivity (kg·kg^-1^)	
	2019	2020	2019	2020	2019	2020
P_1_G_0_	11.41 ± 1.14bc	10.52 ± 1.64c	1.51 ± 0.30c	1.51 ± 0.11d	22.93 ± 0.30c	23.46 ± 0.11c
P_1_G_1_	18.64 ± 1.00a	29.71 ± 3.37a	2.60 ± 0.20b	2.56 ± 0.16b	24.02 ± 0.20b	24.50 ± 0.16b
P_1_G_2_	21.15 ± 0.92a	25.59 ± 3.06a	3.59 ± 0.14a	3.30 ± 0.22a	25.00 ± 0.14a	25.24 ± 0.22a
P_2_G_0_	8.22 ± 1.22c	10.45 ± 1.51c	0.89 ± 0.07d	0.83 ± 0.11e	11.60 ± 0.07f	11.80 ± 0.11f
P_2_G_1_	12.14 ± 2.65b	15.49 ± 1.17b	1.89 ± 0.08c	1.85 ± 0.06c	12.60 ± 0.08d	12.82 ± 0.06d
P_2_G_2_	8.43 ± 1.61c	7.36 ± 3.46c	1.44 ± 0.30c	1.49 ± 0.10d	12.15 ± 0.30e	12.46 ± 0.10e
Significance (*P* value)						
P	67.294*	374.395**	135.870**	43.075**	121.497**	74.359**
GA_3_	16.262**	33.396**	57.190**	84.012**	57.190**	39.546**
P×GA_3_	10.170**	23.436**	21.592**	20.916**	21.592**	18.906**

Different small letters indicate significant difference among treatments (P<0.05). * indicate significant difference at 0.05 level, ** indicate highly significant difference at 0.01 level, the number before * represents the coefficient of variation.

### 3.7 Correlation analysis of different indexes under different phosphorus and GA_3_ treatments

**Figure 5:**
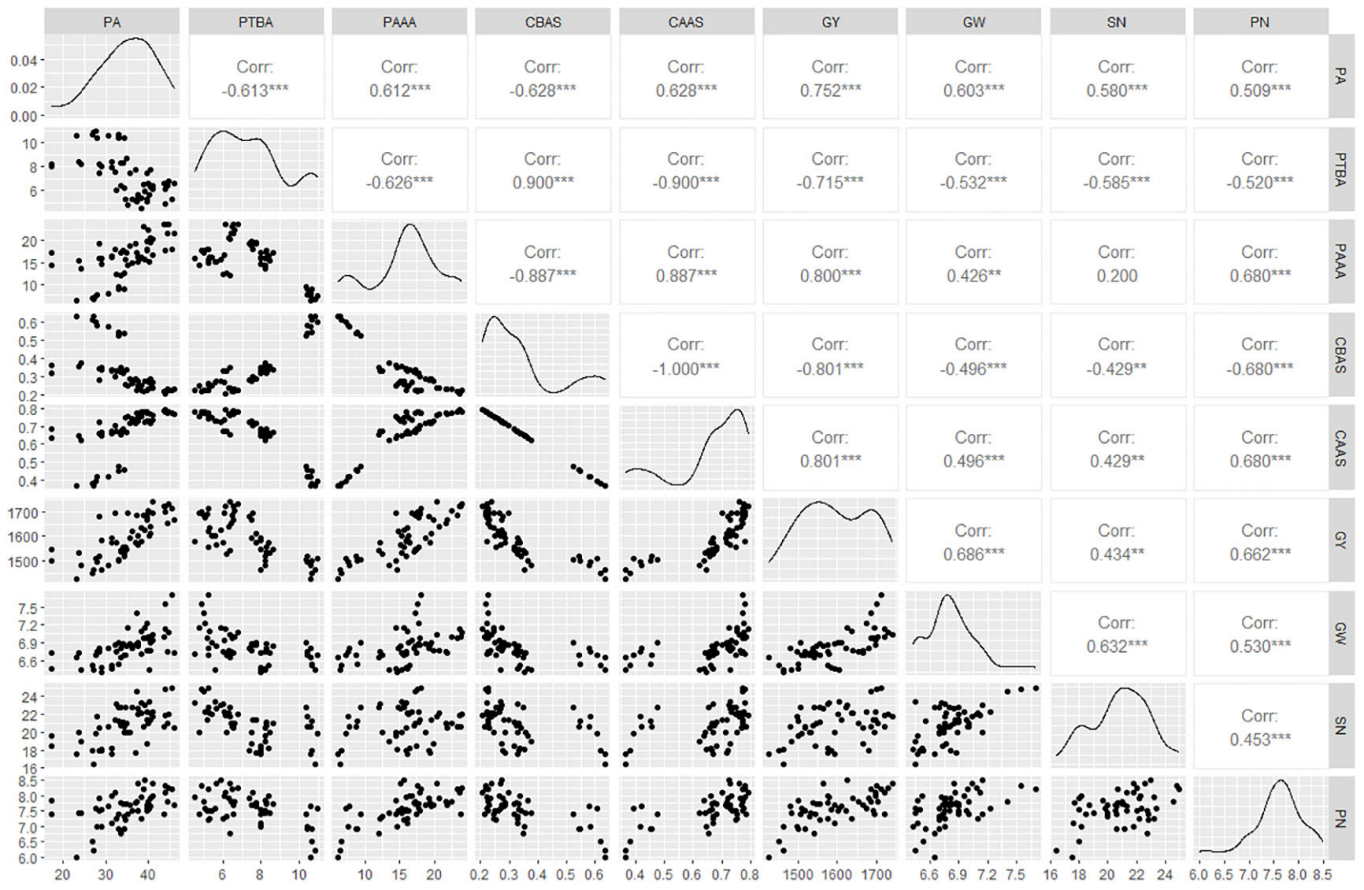
**(A)** Correlation analysis between leaves ACPase activity, phosphorus accumulation and transport. **(B)** Correlation analysis between phosphorus accumulation, transport, and grain yield and yield components. BSB/ASB.1/ASB.3/ASB.5/ASB.10, leaves ACPase activity before spraying/1 d/3d/5 d/10 d after spraying GA_3_ at budding stage; BSA/ASA.1/ASA.3/ASA.5/ASA.10, leaves ACPase activity before spraying/1 d/3 d/5 d/10 d after spraying GA_3_ at anthesis stage; PA, Phosphorus accumulation; PTBA, Phosphorus transfer before anthesis; PAAA, Phosphorus accumulation after anthesis; CBAS, Contribution to grain before anthesis; CAAS, Contribution to grain after anthesis; GY, Grain yield; GW, 1000-kernel weight; SN, Grain number per capsule; PN, Capsule number per plant. * indicate significance at 0.05 level; ** indicate significance at 0.01 level; *** indicate significance at 0.001 level.

Phosphorus accumulation was positively correlated with leaves ACPase activity at 5 and 10 days after spraying GA_3_ at the budding stage and at 1 day after spraying GA_3_ at the anthesis stage. There were also highly significant positive correlations (*P*<0.001) between phosphorus accumulation and phosphorus absorption after anthesis and its contribution to grains, with 0.612 and 0.628 correlation coefficient, which reached a substantial level (**Figure 5A**). The correlation analysis between phosphorus accumulation and transport in oilseed flax plants and grain yield and its components (**Figure 5B**) revealed that the amount of phosphorus absorption and its contribution rate to grain after anthesis were highly significantly positively correlated with grain yield, TKW, and grain number per capsule, with correlation coefficients of 0.800, 0.426, 0.680; and 0.801, 0.496, 0.680, which reached very high, moderate, and substantial, respectively. These results showed that increased the leaves ACPase activity was conducive to phosphorus accumulation of oilseed flax, as well as phosphorus accumulation after anthesis and its contribution to grain, laying the foundation for a high grain yield.

## 4 Discussion

ACPase is a very important inducible enzyme found in plants, and its activity can be used to evaluate phosphorus efficiency (Wasaki et al., 2009; Maseko and Dakora, 2019). The ACPase in the plant can not only convert organic phosphorus in the plant into inorganic phosphorus, but also transport phosphorus in the plant from senescent tissue to young tissue (Ascencio, 2015).And phosphorus absorption and utilization are closely related to the ACPase activity in the plant. When crop plants are subjected to phosphorus stress, the ACPase activity in the plant is greatly enhanced, thus speeding up phosphorus metabolism, promoting organic phosphorus hydrolysis, facilitating phosphorus transport and promoting phosphorus reuse (Shen et al., 2011; Ascencio, 2015). The findings revealed that at P_0_ level, the ACPase activity of leaves responded to the low phosphorus environment in order to increase external phosphorus acquisition and maintain plant growth. While P_1_ significantly decreased ACPase activity of oilseed flax leaves, indicating a negative correlation between soil phosphorus supply and ACPase activity in oilseed flax. At P_2_ level, ACPase activity of leaves increased significantly, indicating that the massive supply of phosphorus could also promote its activity, which was consistent with previous research results (Liu et al., 2022). This study also found that spraying GA_3_ on the basis of applying phosphorus fertilizer (P_1_G_2_ and P_2_G_1_) significantly improved the ACPase activity of oilseed flax leaves, which was conducive to accelerating phosphorus transport and then promoting phosphorus reuse. It may be due to that spraying GA_3_ enhanced photosynthetic rate of oilseed flax, enriched the ACPase distribution in leaves cells, promoted the transportation of available phosphorus from old leaves to new leaves, realized the reuse of available phosphorus, and all of which laid a foundation for the improvement of phosphorus utilization efficiency (Khan and Mohammad, 2013; Sidhu et al., 2018). And no significant difference in hormone levels between 3-5 days after spraying GA_3_ during the 2020 growth season may be due to the decrease of hormone concentration caused by rainfall after GA_3_ spraying.

Phosphorus application is an important agricultural measure for increasing and stabilizing oilseed flax grain yield. Previous research has shown that proper phosphorus application can promote phosphorus accumulation of oilseed flax, but an excessive supply of phosphorus fertilizer will reduce phosphorus accumulation in the later growth stage and cause greater losses (Wu et al., 2016). In this study, the increase of phosphorus fertilizer significantly increased phosphorus accumulation during the whole growth period of oilseed flax. Phosphorus accumulation increased with the increase of phosphorus application at the vegetative growth stage, and it did not differ significantly between P_1_ and P_2_ treatments when entering the reproductive growth stage. This is mainly because an excessive supply of nutrients led to excessive nutrient accumulation before anthesis stage of crops, which increased the negative effect of transportation, and result in a decrease in nutrient accumulation during late growth period (Zhang et al., 2020). While stimulating plant growth, exogenous GA_3_ can accelerate the synthesis of nucleic acid, phospholipid and other biological macromolecules in the plant, improve the activity of enzymes, and thus promote nutrients absorption and utilization (Tuna et al., 2008). This study discovered that the phosphorus accumulation of oilseed flax under the interaction treatment of P_1_G_2_ and P_2_G_1_ during anthesis to maturity period was significantly higher than that of P_0_G_0_.The above results indicated that increasing GA_3_ spraying concentration can effectively alleviate the decline of nutrient accumulation caused by single application of phosphorus in the late growth period of plants, which is consistent with the research results by Wang et al. (2016) on flue-cured tobacco (*Nicotiana tabacum* L.).

The difference of the phosphorus accumulation in the vegetative body at anthesis stage and maturity stage can be used to calculate the phosphorus transport capacity of the vegetative organs of oilseed flax. The phosphorus transport capacity is one of the key markers to measure how much the phosphorus in the vegetative body can transfer to the grain. It is emphasized that the medium phosphorus treatment can significantly improve the phosphorus transport capacity, transport rate and contribution rate to grains, which is conducive to the redistribution of phosphorus in various organs, thus promoting the increase of grain yield (Xie et al., 2014a). In this experiment, phosphorus application significantly increased phosphorus accumulation and its contribution to the grain after anthesis, and spraying GA_3_ could further promote phosphorus transport to the grain. From the perspective of phosphorus fertilizer combined with spraying GA_3_, the phosphorus accumulation and its contribution rate to the grain of oilseed flax after anthesis were higher under P_1_G_2_ and P_2_G_1_ treatments in both growth seasons. It can be seen that the amount of phosphorus and GA_3_ spraying concentration both had an impact on the phosphorus accumulation and transport of oilseed flax. GA_3_ plays a function in promoting phosphorus metabolism and redistribution, and spraying GA_3_ on the basis of phosphorus application can improve the phosphorus accumulation after anthesis and its contribution rate to the grain (Ullah et al., 2017).

The amount of fertilization applied, nutrient accumulation and yield level all have a significant impact on fertilizer utilization efficiency, agronomic efficiency and partial productivity (Chen et al., 2016; Song et al., 2017). The study discovered that crop yield and phosphorus absorption increased with the increase of phosphorus, while phosphorus fertilizer utilization efficiency decreased (Zhu et al., 2012). Exogenous GA_3_ can alleviate the inhibition of external environment on plant growth and nutrient absorption, as well as enhance the growth-promoting effect of fertilizer, and improve nutrient absorption and utilization (Ullah et al., 2022). In this study, the apparent utilization rate, agronomic utilization rate and partial productivity of phosphorus decreased with the increase of phosphorus application rate, which is consistent with previous research results (Wang et al., 2015). Spraying GA_3_ at each phosphorus application level could significantly improve the phosphorus utilization rate of oilseed flax and reduce the phosphorus loss. For phosphorus nutrient management and reasonable phosphorus application in farmland, while taking into account yield, benefit and fertilizer utilization, it is important to explore the change features of soil available phosphorus content and phosphorus profit and loss. This study discovered that the soil phosphorus was deficient when no phosphorus was applied. The surplus amount of soil phosphorus at P_2_ level was much higher than absorption of oilseed flax. Spraying GA_3_ increased the phosphorus deficit when no phosphorus was applied. Applying phosphorus combined with spraying GA_3_ (P_1_G_2_) could significantly reduce the excess phosphorus in the soil. This may be due to that spraying GA_3_ could significantly promote the elongation of oilseed flax’s main root, increase the quantity and density of lateral roots and root diameter, so as to improve phosphorus absorption efficiency (Zhang et al., 2019).

Many studies have reported the effect of fertilizer and exogenous hormone application on crop grain yield (Zhu et al., 2012; Anthony et al., 2013; Nazeer et al., 2020). Research on maize showed that nitrogen application combined with spraying GA_3_ could significantly increase the number of grains per ear and TKW, resulting in a significant increase in maize grain yield (Ullah et al., 2022). Similar studies have also been conducted on ramie (*Boehmeria nivea* L.) (Ullah et al., 2017). In this study, increasing phosphorus fertilizer significantly increased TKW and grain number per capsule of oilseed flax, spraying GA_3_ significantly increased capsule number per plant and TKW. And the interaction effect of phosphorus and GA_3_ on TKW was also significant, ultimately manifested as a significantly higher grain yield under P_2_G_1_ and P_1_G_2_ treatments than other treatments, which is consistent with Khan’s research results (Khan and Mohammad, 2013). The analysis of correlation and correlation degree between oilseed flax grain yield and yield components further confirmed that the main reason for the increase of grain yield was that phosphorus and exogenous hormones promoted the synergistic improvement of yield component factors.

## 5 Conclusions

The interaction of 67.5 kg P_2_O_5_·ha^-1^ and 30 mg·L^-1^ GA_3_ (P_1_G_2_) significantly increase the ACPase activity of oilseed flax leaves and phosphorus accumulation during the whole growth period. And it also promoted the phosphorus accumulation after anthesis and its contribution to grain, thereby increasing the apparent utilization rate, agronomic utilization rate and partial productivity of phosphorus fertilizer, and meanwhile reducing phosphorus fertilizer loss. In terms of grain yield, 67.5 kg P_2_O_5_·ha^-1^ combined with 30 mg·L^-1^ GA_3_ (P_1_G_2_) and 135 kg P_2_O_5_·ha^-1^ combined with 15 mg·L^-1^ GA_3_ (P_2_G_1_) significantly increased capsule number per plant, grain number per capsule and TKW of oilseed flax, and then increased grain yield, reaching 1696 kg·ha^-1^ and 1716 kg·ha^-1^ across two years, respectively. To summarize, 67.5 kg·ha^-1^ phosphorus application combined with 30 mg·L^-1^ GA_3_ spraying can be used as a high-yield and efficient fertilization technology for oilseed flax in the trial area and other similar ecological areas.

## Data availability statement

The original contributions presented in the study are included in the article/supplementary material. Further inquiries can be directed to the corresponding author.

## Author contributions

YZW: Data curation, Writing – original draft. ZC: Data curation, Writing – review & editing. YHG: Writing – review & editing. BW: Writing – review & editing. JYN: Writing – review & editing. BY: Writing – review & editing. YFW: Writing – review & editing. ZJC: Writing – review & editing. MW: Writing – review & editing. PX: Writing – review & editing. HDW: Writing – review & editing. XKM: Writing – review & editing.

## References

[B1] Anthony P. Malzer G. L. Sparrow S. Zhang M. K. (2013). Corn and soybean grain phosphorus content relationship with soil phosphorus, phosphorus fertilizer, and crop yield. Commun. Soil Sci. Plant Anal. 44, 1056–1071. doi: 10.1080/00103624.2012.750337

[B2] Ascencio J. (2015). “Acid phosphatase kinetics as a physiological tool for assessing crop adaptability to phosphorus deficiency,” in Plants for the future. (London: InTech), 79–96. doi: 10.5772/60975

[B3] Bao S. D. (2000). Soil agro chemical analysis. 3rd Edition (Beijing: China Agriculture Press Co., Ltd).

[B4] Bouton S. Leydecker M. T. Meyer C. Truong H. N. (2002). Role of gibberellins and of the RGA and GAI genes in controlling nitrate assimilation in Arabidopsis thaliana. Plant Physiol. Biochem. 40, 939–947. doi: 10.1016/S0981-9428(02)01453-5

[B5] Chen Z. M. Wang H. Y. Liu X. W. Lu D. J. Zhou J. M. (2016). The fates of 15N-labeled fertilizer in a wheat–soil system as influenced by fertilization practice in a loamy soil. Sci. Rep. 6, 1–8. doi: 10.1038/srep34754 27713476 PMC5054427

[B6] Fageria N. K. Filho M. P. B. (2007). Dry-matter and grain yield, nutrient uptake, and phosphorus use-efficiency of lowland rice as influenced by phosphorus fertilization. Commun. Soil Sci. Plant Anal. 38, 1289–1297. doi: 10.1080/00103620701328537

[B7] Falcioni R. Moriwaki T. Benedito E. Bonato C. M. Souzav L. A. Antunes W. C. (2018). Increased gibberellin levels enhance light capture efficiency in tobacco plants and promote dry matter accumulation. Theor. Exp. Plant Physiol. 30, 235–250. doi: 10.1007/s40626-018-0118-1

[B8] Fosu-Mensah B. Y. Mensah M. (2016). The effect of phosphorus and nitrogen fertilizers on grain yield, nutrient uptake and use efficiency of two maize (*Zea mays* L.) varieties under rain fed condition on Haplic Lixisol in the forest-savannah transition zone of Ghana. Environ. Res. 5, 5–22. doi: 10.1186/s40068-016-0073-2

[B9] Grant C. A. Monreal M. A. Irvine R. B. Mohr R. M. McLaren D. L. Khakbazan M. (2009). Crop response to current and previous season applications of phosphorus as affected by crop sequence and tillage. Can. J. Plant Sci. 89, 49–66. doi: 10.4141/CJPS07178

[B10] Kakiuchi J. Kamiji Y. (2015). Relationship between phosphorus accumulation and dry matter production in soybeans. Plant Prod. Sci. 18, 344–355. doi: 10.1626/pps.18.344

[B11] Khan A. U. Lu G. Y. Ayaz M. Zhang H. T. Wang R. J. Lv F. L.. (2018). Phosphorus efficiency, soil phosphorus dynamics and critical phosphorus level under long-term fertilization for single and double cropping systems. Agric. Ecosyst. Environ. 256, 1–11. doi: 10.1016/j.agee.2018.01.006

[B12] Khan M. N. Mohammad F. (2013). Interactive Effect of GA_3_, N and P ameliorate growth, seed and fibre yield by enhancing photosynthetic capacity and carbonic anhydrase activity of linseed: a dual-purpose crop. J. Integr. Agric. 12, 1183–1194. doi: 10.1016/S2095-3119(13)60443-8

[B13] Khan N. A. Mir R. Khan M. Javid S. Samiullah (2002). Effects of gibberellic acid spray on nitrogen yield efficiency of mustard grown with different nitrogen levels. Plant Growth Regul. 38, 243–247. doi: 10.1023/A:1021523707239

[B14] Lin X. Wang D. Gu S. B. White P. Han K. Zhou J.. (2016). Effect of supplemental irrigation on the relationships between leaf ABA concentrations, tiller development and photosynthate accumulation and remobilization in winter wheat. Plant Growth Regul. 79, 331–343. doi: 10.1007/s10725-015-0137-8

[B15] Liu H. Li S. S. Qiang R. W. Liu E. J. Li C. L. Zhang J. J.. (2022). Response of soil microbial community structure to phosphate fertilizer reduction and combinations of microbial fertilizer. Front. Environ. Sci. 10. doi: 10.3389/fenvs.2022.899727

[B16] Ma W. Q. Ma L. Li J. H. Wang F. H. Sisák I. Zhang F. S. (2011). Phosphorus flows and use efficiencies in production and consumption of wheat, rice, and maize in China. Environ. Toxicol. Risk Assess. 84, 814–821. doi: 10.1016/j.chemosphere.2011.04.055 21570104

[B17] Maseko S. T. Dakora F. D. (2019). Relationship between acid phosphatase activity and P concentration in organs of Cyclopia and Aspalathus species, and a non-legume of the Cape Floristic Region. J. Plant Ecol. 2, 12. doi: 10.1093/jpe/rty032

[B18] Nazeer A. Hussain K. Hassain A. Nawaz K. Bashir Z. Ali S. S.. (2020). Influence of foliar applications of IAA, NAA and GA_3_ on growth, yield and quality of pea (*Pisum sativum* L.). Indian J. Agric. Sci. 54, 699–707. doi: 10.18805/IJARe.A-509

[B19] Pan S. G. Rasul F. Li W. Tian H. Mo Z. W. Duan M. Y.. (2013). Roles of plant growth regulators on yield, grain qualities and antioxidant enzyme activities in super hybrid rice (Oryza sativa L.). Rice 6, 9–10. doi: 10.1186/1939-8433-6-9 24280625 PMC4883720

[B20] Shahzado N. Shahmir A. K. Amjad A. Amanullah M. Sajjad R. Muneer A.. (2016). Effect of different levels of phosphorus and method of application on the growth and yield of wheat. Nat. Sci. 8, 305–314. doi: 10.4236/ns.2016.87035

[B21] Shen J. B. Yuan L. X. Zhang J. L. Li H. G. Bai Z. H. Chen X. P.. (2011). Phosphorus dynamics: from soil to plant. Plant Physiol. 156, 997–1005. doi: 10.1104/pp.111.175232 21571668 PMC3135930

[B22] Siddiqui M. H. Khan M. N. Mohammad F. Khan M. M. A. (2010). Role of nitrogen and gibberellin (GA_3_) in the regulation of enzyme activities and in osmoprotectant accumulation in brassica juncea l. under salt stress. J. Agron. Crop Sci. 194, 214–224. doi: 10.1111/j.1439-037X.2008.00308.x

[B23] Sidhu S. K. Kaur J. Singh S. Grewal S. K. Singh M. (2018). Variation of morpho-physiological traits in geographically diverse pigeonpea germplasm under different phosphorus conditions. J. Plant Nutr. 41, 1321–1332. doi: 10.1080/01904167.2018.1450423

[B24] Song K. Xue Y. Zheng X. Q. Lv W. G. Qiao H. X. Qin Q.. (2017). Effects of the continuous use of organic manure and chemical fertilizer on soil inorganic phosphorus fractions in calcareous soil. Sci. Rep. 7, 1164. doi: 10.1038/s41598-017-01232-2 28442726 PMC5430788

[B25] Tuna A. L. Kaya C. Dikilitas M. Higgs D. (2008). The combined effects of gibberellic acid and salinity on some antioxidant enzyme activities, plant growth parameters and nutritional status in maize plants. Environ. Exp. Bot. 62, 1–9. doi: 10.1016/j.envexpbot.2007.06.007

[B26] Ullah S. Anwar S. Rehman M. Khan S. Zafar S. Liu L. J.. (2017). Interactive effect of gibberellic acid and NPK fertilizer combinations on ramie yield and bast fibre quality. Sci. Rep. 7, 1–9. doi: 10.1038/s41598-017-09584-5 28878353 PMC5587721

[B27] Ullah I. Dawar K. Tariq M. Sharif M. Fahad S. Adnan M.. (2022). Gibberellic acid and urease inhibitor optimize nitrogen uptake and yield of maize at varying nitrogen levels under changing climate. Environ. Sci. pollut. Res. 29, 6568–6577. doi: 10.1007/s11356-021-16049-w 34455561

[B28] Wang J. W. Li H. J. Huang X. L. Hu W. Wang S. H. Zhou Z. G. (2023). Phosphorus application affected cottonseed kernel yield and nutritional quality by improving oil yield and quality of two cotton (*Gossypium hirsutum* L.) cultivars differing in phosphorus sensitivity. Field Crops Res. 291, 108778. doi: 10.1016/j.fcr.2022.108778

[B29] Wang Q. Y. Tian J. M. Han X. Huang T. R. Yang Q. F. Tang D. B.. (2015). Effect of phosphorus on yield and nutrient absorption and utilization in starch-type sweet potato. Plant Nutr. Fert. Sci. 21, 1252–1260. doi: 10.11674/zwyf.2015.0519

[B30] Wang L. Zhu J. F. Xu Z. C. (2016). Interactive effects of exogenous hormone on quality of middle leaves among flue-cured tobacco after the time of topping. J. Nucl. Agric. Sci. 30, 2411–2417. doi: 10.11869/j.issn.100-8551.2016.12.2411

[B31] Wasaki J. Maruyama H. Tanaka M. Yamamura T. Dateki H. Shinano T.. (2009). Overexpression of the *LASAP2* gene for secretory acid phosphatase in white lupin improves the phosphorus uptake and growth of tobacco plants. Soil Sci. Plant Nutr. 55, 107–113. doi: 10.1111/j.1747-0765.2008.00329.x

[B32] Wu B. Gao Y. H. Li Y. Yan B. Cui Z. J. Zhang Z. K.. (2016). Oil flax phosphorous transformation, distribution and utilization under nitrogen phosphorous combination on dry land. Oil Crop Sci. 38, 619–625. doi: 10. 7505/j. issn.1007-9084. 2016.05.012

[B33] Xie Y. P. Li A. R. Yan Z. L. Niu J. Y. Sun F. X. Yan B.. (2014a). Effect of different phosphorus levels on phosphorus nutrient uptake, transformation and phosphorus utilization efficiency of oil flax. Acta Prataculturae Sin. 23, 158–166. doi: 10.11686/cyxb20140119

[B34] Xie Y. P. Niu J. Y. Gan Y. T. Gao Y. H. Li A. R. (2014b). Optimizing phosphorus fertilization promotes dry matter accumulation and P remobilization in oilseed flax. Crop Sci. 54, 1729–1736. doi: 10.2135/cropsci2013.10.0672

[B35] Yang W. B. Ni Y. L. Cai T. Ni Y. L. Guo J. X. Peng D. L.. (2012). Effects of exogenous abscisic acid and gibberellic acid on filling process and nitrogen etabolism haracteristics in wheat grains. Aust. J. Crop Sci. 7, 58–65. Available at: https://www.cropj.com/yang_7_1_2013_58_65.pdf.

[B36] Zhang L. Du J. Kong L. L. Yin C. X. Zhao Y. K. Hou Y. P.. (2020). Effects of phosphorus fertilizer application on yield, phosphorus absorption and utilization, soil phosphorus balance of spring maize in black soil region of northeast China. J. Northeast Agric. Univ. 45, 38–42. doi: 10.16423/j.cnki.1003-8701.2020.05.010

[B37] Zhang X. R. Wang B. M. Zhao Y. J. Zhang J. R. Li Z. X. (2019). Auxin and GA signaling play important roles in the maize response to phosphate deficiency. Front. Plant Sci. 283, 177–188. doi: 10.1016/j.plantsci.2019.02.011 31128687

[B38] Zhu X. K. Li C. Y. Jiang Z. Q. Huang L. L. Feng C. N. Guo W. S.. (2012). Responses of phosphorus use efficiency, grain yield, and quality to phosphorus application amount of weak-gluten wheat. J. Integr. Agric. 11, 1103–1110. doi: 10.1016/S2095-3119(12)60103-8

